# Environmental Methamphetamine Exposures and Health Effects in 25 Case Studies

**DOI:** 10.3390/toxics8030061

**Published:** 2020-08-20

**Authors:** Jackie Wright, Michaela Kenneally, Kirstin Ross, Stewart Walker

**Affiliations:** 1 College of Science and Engineering, Flinders University, GPO Box 2100, Adelaide 5001, Australia; kirstin.ross@flinders.edu.au (K.R.); stewart.walker@flinders.edu.au (S.W.); 2 Environmental Risk Sciences Pty Ltd., P.O. Box 2537, Carlingford Court 2118, Australia; 3 Forensic Science SA, GPO Box 2790, Adelaide 5001, Australia; Michaela.Kenneally@sa.gov.au

**Keywords:** methamphetamine, environmental exposure, hair analysis, health effects, use, manufacture

## Abstract

The clandestine manufacture and use of methamphetamine can result in contamination of residential properties. It is understood that this contamination remains in homes for a significant period, however there are a lack of data available to understand the health effects of exposure to environmental methamphetamine contamination (third-hand exposure). Our study collected information from 63 individuals in 25 separate case studies where the subjects had unwittingly suffered third-hand exposure to methamphetamine from former manufacture, use, or both. Data included environmental contamination data, information on subjects’ health effects, and evidence of exposure using hair analysis. This study identified a range of health effects that occur from residing in these properties, including behavioural effects or issues, sleep issues, respiratory effects, skin and eye effects, and headaches. Methamphetamine was detected in hair samples from some individuals, including children. The exposures and concomitant reported health effects covered a wide range of environmental methamphetamine levels in the properties, including low levels close to the current Australian guideline of 0.5 µg methamphetamine/100 cm^2^. There were no discernible differences between health effects from living in properties contaminated from former manufacture or use. This study demonstrates that residing in these properties can represent a serious public health risk.

## 1. Introduction

Unlike the controlled manufacture of drugs, the clandestine manufacture of methamphetamine results in the uncontrolled storage, use, generation, and disposal of a wide range of chemicals, as well as the deposition of methamphetamine drug residues on indoor surfaces [1]. Further, the smoking of methamphetamine results in the deposition of residues onto the surfaces of properties where the activity is undertaken [2,3]. These residues have been found at varying levels on a wide range of household surfaces and materials, and have been shown to persist for months or even years [1,4,5,6]. People working or living in these contaminated properties are exposed to these methamphetamine residues. At present, there is limited information available in relation to these exposures and the health effects that occur as a result [7,8].

The operation of clandestine methamphetamine laboratories results in the presence of a wide range of hazards and risks within the premises during and following manufacture [7]. The most significant adverse health effects are those derived from immediate acute hazards. These hazards include the uncontrolled and unprotected storage and use of chemical precursors that are volatile, flammable, or reactive; and the release of high concentrations of toxic gases (that may include ammonia or phosphine) into a room or home where ventilation is limited and where there is the potential for unprotected exposures. Data are available [9,10,11,12,13,14] that show that a range of individuals, including children, in clandestine drug laboratories are at high risk of injury and illness associated with immediate hazards such as fires, explosions, and chemical incidents, as well as acute and chronic exposure to the range of chemicals used to manufacture the drugs and the drugs themselves. There is also a significant body of research related to the health effects of methamphetamine use, including the effects of exposure of children to methamphetamine in utero [15,16,17].

However, once manufacture and use has ceased and the acute hazards associated with manufacture are no longer present, methamphetamine contamination remains at the property. If this contamination is not remediated it can remain for a long period of time [6], resulting in long-term or chronic environmental (also known as third-hand) exposure to people living or working in these properties. The evidence suggests that the level of methamphetamine manufacture and use in Australia is increasing. It is estimated that police only detect 1 in every 10 drug laboratories [18,19]. Guidance is available in Australia for the assessment and remediation of former clandestine drug laboratories, however there are a large number of properties contaminated as a result of manufacture and use where the presence of contamination is unknown [3], and there are no regulatory requirements for the testing and remediation of these properties. Hence, in most situations, exposure to methamphetamine residues in properties purchased or rented by the public is unknown.

It is commonly assumed that third-hand exposure to drugs such as methamphetamine is low compared with exposure related to illicit drug use, exposures during manufacture, or legal therapeutic use of amphetamine-based drugs, with the likelihood of adverse health effects assumed to be negligible. This assumption is principally due to the lack of data available on the health effects of environmental methamphetamine exposures.

This study has been undertaken to characterise and better understand exposures and health effects associated with unwitting environmental exposures to methamphetamine residues in residential properties.

## 2. Materials and Methods

### 2.1. General

The focus of this study related to characterising exposures and health effects in individuals who have had unwitting environmental exposures to methamphetamine contamination as a result of residing in place of former manufacture or use. These individuals are difficult to identify, as the activities and behaviours that result in the presence of methamphetamine contamination in properties are illegal, and therefore are unknown to those purchasing or renting a property. As a result, the information and data that can be obtained in relation to these exposures is derived from opportunistic case studies.

The case studies presented in this research were obtained over the period 2013 to 2019 and provide varying amounts of concomitant data on exposure, characterised on the basis of environmental contamination levels, biological data, health effects, and interview data. As the case studies are opportunistic, the information and data obtained relate to varying time periods and exposure situations.

All individuals gave their informed consent for inclusion prior to participation in the study. Ethics approval for all case studies included in this research, to collect information on environmental contamination levels, to conduct interviews, to evaluate behavioural issues in children using the Behavior Assessment System for Children (BASC), Parent Rating Scheme (PRS) as BASC-2:PRS and BASC-3:PRS [20,21], and to obtain hair samples, where relevant, was obtained from the Southern Adelaide Clinical Human Research Ethics Committee (Application 477.11, approved 15 March 2012 with extension approved 27 October 2015) and the Social and Behavioural Research Ethics Committee (SBREC) at Flinders University (Project No. 7902, approved 14 March 2018).

### 2.2. Identification of Opportunistic Case Studies

These case studies were identified through links with contamination assessment and remediation companies, legal firms, and real estate organisations who identified individuals and families living in methamphetamine-contaminated homes and recommended their participation in this study. Individuals identified from these opportunistic case studies were only included if these individuals had resided in properties formerly known or suspected to have been used for the manufacture or smoking of methamphetamine. Current users of methamphetamine and other amphetamine-type stimulants were identified on the basis of responses to questions on drug use and were not included in the study. Once identified, informed consent was obtained for all individuals who participated in this study.

### 2.3. Characterisation of Exposure

Potential exposures within each property were evaluated on the basis of two types of data: environmental contamination levels and hair analysis.

#### 2.3.1. Environmental Contamination Levels

The presence of methamphetamine contamination in properties was characterised on the basis of surface wipe testing. These data are collected and are inferred to represent the mass of methamphetamine on different surfaces that may be accessible and available for participants to come into direct contact with, and where these residues may transfer onto the skin and other objects and ingested or dermally absorbed. Data relating to the nature and extent of methamphetamine contamination in each property was provided by the property owner or tenant, as the result of environmental testing conducted by an independent testing company. Data provided for use in this study relates to individual wipe samples collected from each premises, and analysed by various accredited laboratories for methamphetamine, along with amphetamine, ephedrine and pseudoephedrine (in most cases). The data provided have been reviewed to ensure compliance with NIOSH guidance in relation to sampling [22].

#### 2.3.2. Hair Analysis

Where participants had been exposed in a property for more than three months and participation in this study occurred during or just after the period of exposure occurred, hair samples were collected (under informed consent protocols) to provide an estimate of environmental intake or exposure.

The hair samples were collected using a hair sampling kit in accordance with the hair sampling procedure provided by Forensic Science SA, Adelaide, Australia. This procedure is consistent with the sample collection procedure detailed in the Society of Hair Testing guidelines [23]. This involved giving the sample a unique identify code, using gloves, and cutting hair from the crown or vertex of the head as close to the scalp as possible using clean sharp scissors. Where possible, the hair sampled was approximately the thickness of a pencil. Where the hair was thin or short, hair was sampled from more than 1 location on the crown or vertex to maximise the amount of hair sampled. Each hair sample was contained in the supplied aluminium foil, ensuring that the cut end was aligned with the marking on the foil and that the foil wrapped around the hair sample. Each hair sample was then placed into a supplied envelope, which was sealed for chain of custody purposes, with the sample ID, date, and time of collection noted. The envelope and copy of the participant’s informed consent form were then placed into a sealable bag, which was then sealed. The samples were securely stored at room temperature prior to analysis. Information relating to hair colour and whether the hair was dyed was recorded.

The hair samples were analysed for methamphetamine and amphetamine by Forensic Science SA. The method involves extraction using methanol and analysis using liquid chromatography with tandem mass spectrometry (LC-MS/MS) using an electrospray ionisation (ESI) source.

*Preparation and Extraction:* An approximately 3 cm segment of hair was cut into segments of 1–5 mm in length, then 20 mg transferred into a glass tube. Any environmental (external) contamination of the sample was removed by a brief (approximately 30 s) wash with 2 mL methanol. The methanol wash was analysed separately. An internal standard (d^5^-methylamphetamine for methylamphetamine or d^5^-amphetamine for amphetamine) was added and extraction of the drugs from the sample was achieved by incubation overnight (approximately 18 h) at 45 °C in 2 mL methanol. Following extraction, the sample was allowed to cool to room temperature and the methanol was transferred via pipette to a disposable test tube. Acid alcohol (20 µL of 0.5% hydrochloric acid in methanol) was added to form the hydrochloride salt of the amphetamines prior to solvent evaporation under a steady stream of nitrogen at 40 °C. This ensures that amphetamines are not lost in the evaporation stage. The residue was reconstituted with 100 µL of 0.1% formic acid to match the mobile phase and ensure satisfactory chromatographic peak shapes and separation. The samples were transferred to a 2 mL vial then capped and centrifuged for 5 min prior to analysis.

*Analysis:* The extract was analysed by LC-MS/MS using an ESI source, as described above. The instrument used was an Agilent 1200 LC system with Applied Biosystems 4000Q-Trap MS. The column was a Phenomenex Luna PFP(2) 3 µm 50 × 4.6 mm with a pentafluorophenyl (PFP) guard column measuring 5 µm 4 × 2.0 mm. Deuterated analogues of the drugs were used as internal standards. A blank and quality control samples (purchased commercial external hair controls and a previous drug-positive proficiency case sample) were included with each batch run. Calibration curves were constructed and used to calculate the drug concentrations in the samples.

The sensitivity of the instruments enables the identification and quantification of trace levels of drugs, with a quantitation limit of 5 pg drug per mg hair (pg/mg) for amphetamines. While it is common for the reporting of drugs in hair to include a reporting limit (to remove low level detections that may be the result of prescribed medications or low level instrument error), the analysis undertaken for this study included all trace level detections, as none of the participants were drug users.

### 2.4. Characterisation of Health Effects

#### 2.4.1. Interview Data

Participants in the study were interviewed. Where children were involved, information was provided by the parent or caregiver. Each participant was given a unique ID, which was used for all information relating to the participant, including the hair analysis (where relevant). The interview involved a number of questions that related to the following:

- Participants—age, gender, hair colour, whether the hair was dyed, use of amphetamine-type stimulants, including attention-deficit/hyperactivity disorder (ADHD) drugs (particularly relevant for children);
- Housing situation—owning or renting, duration of time at the property, how much time is spent in the property, including whether the participant was the primary cleaner in the home, if they had undertaken renovations in the home, or whether children undertake a lot of floor play;
- Exposure situation—how the participant or family came to be living in the property and how they found out that the property was contaminated. Information about whether the property may have been contaminated from manufacture or use was also obtained;
- Health information—identification of pre-existing conditions, description of health issues that occurred while living in the property, with medical records or school attendance plus medical records provided to support the information provided, and information on whether the health effects persisted when out of the property. Where the health problems had resolved, questions relating to how long the health effects persisted after moving out of the home were also included. The collection of health information focused on health effects that occurred within the property that were different from or worse than health issues experienced prior to living in the property. As the health effects being documented were self-reported, it was considered important to document health effects specifically related to the time spent in the property. For some case studies, children had spent their whole lives living at the contaminated property. For these participants, the health information obtained from parents related to their overall health. In some cases, health effects related to the property could be identified more clearly as these did not persist whenever the participant was out of the property.

#### 2.4.2. Behavioural Assessment

For children involved in the study, a behavioural assessment was completed by the parent or caregiver using the Behaviour Assessment System for Children Second Edition (BASC-2) and Third Edition (BASC-3) [20,21] forms for each child. BASC-2/BASC-3 are standardised assessment tools that provide information to assist in assessing a child’s behavioural, emotional, and adaptive functioning. The tools can be used with children aged from 2 years to adolescents aged 21 years. There are a range of forms and scales available. For the purpose of this assessment, the Parent Rating Scales (PRS) were used. The PRS forms used related to the ages of the children residing at the premises, i.e., aged between 2 and 5 years, between 6 and 11 years, or 12 to 21 years. The scales use four choices for responses to each of the questions asked: never, sometimes, often, and almost always.

Once completed by the parent or caregiver, the BASC-2/BASC-3 forms were scored using the Pearson online scoring system. The online scoring includes a check for the validity of responses and scoring against normalised groups, with the general combined-gender norm group used for the scoring in this study.

## 3. Results

### 3.1. Opportunistic Case Studies

Twenty-five opportunistic case studies were identified and included in this study between 2013 and 2019. As these case studies were opportunistic, each case study was different, particularly in relation to the housing and exposure situations. The following provides a brief summary of the exposure situation relevant to each of the case studies, specifically the individual situations, along with unique characteristics and issues related to each property.

CS01: This case study related to a rural property. Police had seized chemicals and manufacturing equipment from the property and notified the local council of the seizure. The owner of the property at that time was arrested and charged. The local council issued a notice to the owner requiring assessment and remediation, however the notice was not acted on and was not followed up by the council. The property was subsequently sold to a family with 2 adults and 3 children. The new owners were notified by the local council approximately 8 months after moving in that the property was formerly used to manufacture methamphetamine and there was an outstanding notice. The local council undertook an assessment of contamination that took another 10 months, after which time it was clear that the property was contaminated and the family moved out, leaving all possessions behind. The family lived in the contaminated property for approximately 18 months. In relation to this case study, additional testing of the property has been undertaken by the researcher over a number of years, in addition to the data provided by the local council [6].

CS02: This case study related to a rental property in an urban area occupied by a mother and 2 children. At the time of signing the rental agreement, there was no information provided that indicated that the owner of the property had been arrested for the manufacture of methamphetamine. Management of the property was undertaken by the owner’s mother. At the time of rental, the owner’s mother, sister, and letting agent (a friend of the sister) were aware that the property had formerly been used to manufacture methamphetamine, but the tenant was not informed. The owner cleaned the property and replaced curtains prior to letting. Neither the police nor the local council had knowledge of the property in relation to the manufacture of methamphetamine. The tenant became aware of potential methamphetamine contamination as a result of conversations with neighbours, who told them about the former tenant, who received a prison term for methamphetamine manufacture. The tenant was also suspicious of contamination as the tenant’s son’s behaviour was significantly different when living at home compared with times when he spent at least a week out of the home. The tenant had the property tested. Once contamination was discovered and was found to be higher in the lower level (basement) where the tenant’s son resided, the family moved out without their possessions. The local council was notified of the results and they issued a clean-up notice to the owner.

CS03: This case study related to the purchase of a home in an urban area, which was occupied by a single adult. The home was not known to have been formerly used for the manufacture of methamphetamine at the time of purchase, as there was no notification on any searches undertaken during the sale of the property. When the property was purchased, the owner started renovation works and became unwell very soon after commencing these works. The owner became concerned about the property when she saw a newspaper article about police seizing manufacturing equipment and arresting the occupant of another property. She recognised the arrested individual as the former occupant of her property. The owner also notified the local council and was informed that the council was aware the home was formerly used to manufacture methamphetamine, but this information was not provided at the time of property sale. There was also information that the property had some level of remediation (details unknown), however testing after renovations were undertaken indicated that the property had been re-contaminated as a result of renovations.

CS04: This case study involved a short-term private rental of an urban property by a family with 2 adults and 2 young children. Within days of moving into the property the neighbours told them the previous tenant had been involved in manufacturing methamphetamine in another house across the road and was currently involved in court proceedings. The neighbours did not know whether their home had been used to manufacture methamphetamine or whether the tenant had only used methamphetamine in the house. The tenant contacted the owner, who refuted the claim of manufacture and stated the house had been professional cleaned prior to occupancy. In addition, the tenant bleached the walls on moving in. The children were unwell whenever spending time in the house, so the tenants organised to get the property tested. The property was found to be contaminated and the tenants moved out.

CS05: This case study involved the rental of an urban public housing property with a known drug use history by 2 adults and 1 child. The property was initially tenanted by the family’s mother who had a history of drug use, including heavy use (primarily smoking) of methamphetamines. During her time living at the property (more than 10 years), she took in a number of boarders, some of whom were also drug users. The property was affected by methamphetamine contamination primarily as a result of the long-term smoking of the drug. However, there was a suspicion that the property may have also been used for the manufacture of methamphetamine by boarders staying at the property. After moving into the home, all occupants experienced health problems. After suspicions about contamination were raised, the property owner tested the property and determined it was not suitable for occupancy. The family were told to leave the property by the public housing authority, leaving all their personal property in the house.

CS06: This case study involved the rental of an urban public housing property by a single adult for a period of 7 years. Within a few weeks of moving into the home the occupant experienced a range of health problems, which persisted when she was in the home. She became suspicious of the presence of contamination in the home after becoming aware that exposure to methamphetamine contamination can result in similar health effects as being experienced. The property was tested for contamination and found to have levels in excess of the relevant guideline. The tenant vacated the property once the results were received by the owner. When the occupant originally moved in, she noted the presence of drug bags and needles hidden in cupboards and under rugs and loose floor tiles, and people would often approach the house looking to buy drugs. There was no evidence or suspicion of manufacture. This suggested the property had a prior history of drug use (and potentially supply) but not manufacture. It is noted that the property is located directly adjacent to a major motorway where elevated levels of noise and vehicle exhaust fumes are also noted to be an issue. The tenant had significant levels of stress during and after discovering the property was contaminated due to the way in which the issue was handled. Some of the health effects were resolved on moving out, however a number of health effects have remained unchanged for years, in particular eye damage and chronic fatigue.

CS07: This case study involved the rental of an urban home by an adult and one child for a period of 2 years. While living in the home, the daughter’s behaviour changed significantly and the cause could not be identified by her family doctor or from teachers from her school (who had also noted the changes in behaviour). Discussions with neighbours about these problems identified that the previous tenants had a history of drug use and drug dealing behaviour. The landlord had to do a number of repairs to the home before renting it out again, however no assessment or remediation of contamination was undertaken. Subsequent testing of the property identified the presence of methamphetamine contamination, after which time the tenants vacated the property.

CS08: This case study involved a house on the urban outskirts of a major city that was purchased by a family of 2 adults and 3 children. The property was vacant for a number of months prior to the sale. During the sale of the property, access into the property was restricted and the purchasers were advised to change the locks once moved in for their safety. Information from some of the neighbours indicated that the previous occupants used methamphetamine and there was also some suggestion of drug manufacture. Other neighbours refuted the manufacture but confirmed that the previous owners used drugs in the property. Discussions with police did not identify any drug-related reports. After the family moved in, they started renovations, and given the potential drug history at the property they became concerned about contamination and had the property tested. They had noticed some health issues and sleep issues with one of their children. The testing showed the property was contaminated. They lived in the property for 4 months prior to discovering it was contaminated. They could not afford to move out so lived in a caravan on the property until the remediation was completed.

CS09: This case study involved the rental of an urban unit by 2 adults for a period of approximately 5 months. Following concern about the condition of the property being rented, contamination testing was undertaken, which identified the presence of methamphetamine contamination. The tenants were issued with an order to vacate from the owner, citing the property was unsafe for occupancy. The couple renting the property left without their possessions. No health effects were reported by the couple living in the property, however they were expecting a baby and were very concerned about the health of their unborn child, as well as the condition their property would be returned in after remediation (which was proposed). They did not want to have any possessions returned that had any potential for methamphetamine to be present, as many of the items were new baby items. This couple had to take legal action to recover some costs associated with possessions that had to be destroyed. After moving out the couple had a healthy baby.

CS10: This case study involved the rental of a unit in a rural town by a single adult for 3 years. While not a public housing unit, the property was from a charity rental group. The occupant was a victim of domestic violence and needed urgent accommodation, so the property was rented sight unseen. The property was in poor condition and a neighbour indicated that the unit had been used to cook methamphetamine. The occupant became unwell, although she was not aware that exposure to contamination from former methamphetamine cooking could cause health issues until she saw a media article on the issue. Some of the health issues experienced related to mood, anxiety, and depression, and at times she thought she was going mad and attempted suicide. She asked the landlord about the presence of contamination, who initially offered to pay her and fix the outstanding repairs on the property, however this was not accepted, and the landlord ultimately tested the property. The property was found to be contaminated and the occupant moved out without any possessions. Many of the health effects were resolved when the tenant moved out of the property.

CS11: This case study involved the rental of a home by a single adult in a large country town for approximately 7 months. The occupant’s previous rental property had been sold and she was given 30 days to vacate. She had pets, meaning rental options were limited, and this was the only property that would allow pets, so she felt she had no choice with the property. She cleaned the home on moving in but felt unwell whenever in the home. The real estate agent she was dealing with had started testing some rental properties for methamphetamine, so she requested that her property be tested given how unwell she had been. The testing confirmed that the property and her possessions were contaminated and she had to move out, leaving her property behind. The source of contamination (manufacture or use) is not known. The issue had to go through a tenancy tribunal to sort out personal property and get compensation for property that had to be destroyed.

CS12: This case related to the rental of an urban house by a family of 2 adults and 3 children for approximately 4.5 months. The family moved from a rural area to a major city due to a change in employment and rented a property. When they moved into the property, they found a syringe. Neighbours indicated that the previous tenant had 2 large guard dogs and there was often unusual activity at night. The children were noticeably unwell and behaved differently within the house. With this information and awareness of the potential for methamphetamine contamination to be of concern, the tenants had their property tested. The testing showed that the property was contaminated and they moved out of the home, leaving their possessions behind. It is not known whether the contamination was the result of manufacture or use.

CS13: This case study involved exposure by an adult with diagnosed multiple chemical sensitivity to contamination in an urban home. The participant had been living in community housing for the past 20 years and was required to move due to the need for major renovations. He moved into a new community housing property that was observed to be very rundown. Every time he visited the property for short periods of time (up to 40 min duration) while taking possessions to the property, he felt unwell. This only occurred when entering the home, even for short periods of time, with the effects lasting after exiting the property for up to a few days. The adverse health effects tended to become continuous with repeated entry into the home. He was suspicious of past activities in the home, believed to be heavy drug use, based on discussions with neighbours. Awareness of potential methamphetamine contamination prompted him to get the property tested. This identified that the property was contaminated. He never completed moving in, however he had to deal with his possessions that had already been moved in and were found to be contaminated.

CS14: This case study involved exposure of a young child to residues in the mother’s home in an urban area during visitation over a period of just over 2 years. The mother and father were separated and the child was spending equal time between the mother and father. The mother was known to be using methamphetamine in the home when the child was not present. The father was on a methadone program. The father was concerned about the child being exposed to methamphetamine in the home whenever he was residing with the mother. He noticed that the health and behaviour of the child was different or poor whenever the child had spent time at the mother’s property. Testing of the child’s hair identified trace levels or methamphetamine. The presence of methamphetamine in the child’s hair was of concern to the mother and father, and resulted in the mother ceasing methamphetamine use and moving out of the contaminated property. The child’s health has improved significantly.

CS15: This case study involved a mother and child renting an urban community housing unit for approximately 3 years. The family had been living in community housing for a while and transferred to this property following a domestic violence incident. When moving into the unit she noticed that both she and her son’s health were impaired, and initially thought it may be the result of exposure to mould. Discussions with neighbours indicated that the police had arrested the previous tenant (6 months prior to her moving in) on drug-related charges (specifics unknown). The housing authority did not inform her of the previous drug and police history. After thoroughly cleaning the house, heightened awareness through media reports of the potential presence of methamphetamine contamination made her suspicious. Her son’s health was problematic, with significant asthma and constant respiratory infections resulting in him missing a lot of school. This, in turn, made it difficult for the mother to secure employment. She had the property tested, and while methamphetamine was detected, it was not above the relevant guideline. She insisted that the properly be remediated to remove the methamphetamine contamination. The property was remediated, and both her and her son’s health have improved since remediation was completed. The family remains living in the property.

CS16: This case study involved the rental of an urban unit by a single female adult with previously diagnosed multiple chemical sensitivity. She was previously living in a property that was contaminated with mould that made her unwell. She had to move out of that property and leave most of her possessions behind due to the effect it was having on her health. She moved into a different unit that was clean as a short-term rental in order to help her recover from the effects of living in the mould-affected home. A more permanent rental unit became available in the same building and she was told it was the same as the unit she was in and was also clean. She moved out of the short-stay and into the rental property. When she moved into the property, she became unwell again. She experienced a wide range of significant health problems in the unit. She became suspicious of drug activities in the building and in her unit based on the dirt and dust, noting strange smells and items found in the unit (e.g., a bag of pseudoephedrine under tiles on deck). She suspected another unit close to hers was being used for manufacture. As her health declined, her partner had to leave his job to become a fulltime carer. She contacted police with her suspicions of manufacture. She had the property tested for methamphetamine contamination and elevated levels were found. She moved out, leaving her possessions behind. Initially she had to live in her car as she had no money for alternate accommodation. Her health improved over time, taking around 6 months for most of the effects to be resolved.

CS17: This case study involved the rental of a home in a suburban area by a single male adult for a period of approximately 5 months. The property was rented through a rental agency, with no issues identified. The property was clean and tidy. Not long after moving in, a car arrived and the occupants informed him that the property had a drug history. Further discussions with neighbours also indicated that the property had a drug history. Enquiries with the property manager indicated that they knew the previous tenants were methamphetamine users. The real estate agency stated that they were only aware of methamphetamine use and no manufacture was suspected. However, manufacture could not be precluded. Subsequent enquiries with the agent indicated that the agent had previously found a knife taped to the back door, and during a previous inspection they found a table full of drugs and paraphernalia in the garage. On further inspection, the tenant found a packet of red powder hidden in the roof space and the property had a number of unusual water connections in the bathroom, laundry, and kitchen. There were also indications that the windows and some doors had been previously screwed or bolted shut. The tenant worked from home and became generally unwell. The property was tested for methamphetamine contamination, was found to be contaminated, and he moved out of the property without his personal possessions.

CS18: This case study involved the rental of a suburban home by a family of 2 adults and 2 children for a period of approximately 8 months. The family had moved from another city and the availability of rental properties was limited. They rented the property without an inspection, but were told by the estate agency that the property was clean and tidy, which was found not to be the case. The property was in poor condition and messy when they moved in. After they had moved in a sacked staff member from the real estate agency approached them and told them the previous tenant at their rental property was evicted for methamphetamine manufacture, and that the estate agency knew this was the case at the time the property was rented. No cleaning or remediation had been undertaken on the property. All members of the family were unwell in the property, with the mother and younger son more significantly affected. In particular, the mother was hospitalised three times due to severe headaches. Once the tenants were aware of the former drug history of the property, they had the property tested and it was found to be contaminated. They moved out without their possessions.

CS19: This case study involved a family of 2 adults and 2 children who purchased a suburban residential home 8 years ago and subsequently discovered it was contaminated with methamphetamine. Both their children were born while living at the property. Shortly after they had moved into the property, a neighbour told them the property had a drug history, however they dismissed this as unsubstantiated. The property had been well maintained and cleaned over the years. In addition, much of the property had been renovated and painted. Recent awareness of the potential presence of methamphetamine contamination from manufacture or use prompted the family to get the property tested. The initial sampling identified the property was contaminated and they moved out. The family found a newspaper article published about their home prior to their purchase of the home that indicated it was busted by police as a clandestine drug laboratory. The initial testing focused on timber that had not been painted or renovated, although this was not in areas that the family would come into contact with regularly. Further testing of surfaces that the family regularly touched, which had been repainted or were composed of new materials from renovations, was undertaken and little to no contamination was found. This case study did not find that the methamphetamine contamination was mobile or transferable within the property, which differs from many other properties presented here and may be due to the nature of the building materials in the home.

CS20: This case study involved the purchase of a rural property by a couple as a place for retirement. Renovations were being undertaken and the owners stayed in the property for only a few days at a time over a period of approximately 2 years. The property was purchased from a deceased estate. The previous owners had built the home, which was very well presented and clean. The new owners undertook a number of renovations, including painting. Once these works were complete, they spent more time at the home. When they had purchased the house, they asked the real estate agent about any former drug history and were told there was no history. Once they moved in, neighbours informed them that the previous owners were troublesome and involved with drug activities (potentially dealing), and the property was found to have been known to police. Both owners had a number of unexplained health effects since taking possession of the property, in particular the adult male. Once the property was known to be contaminated, they no longer entered the property without PPE. They organised to get the property tested and remediated. The initial testing focused on surfaces that were not renovated or painted by the owners. Further testing was then undertaken on all other surfaces in the property, including some areas where the paint was sanded back to identify levels of contamination beneath the freshly painted surface. This property had a significant number of samples collected from a range of surfaces and areas that the owners would come into direct contact.

CS21: This case study involved the rental of an urban residential home by a family of 2 adults and 1 teenage child for a period of approximately 4 weeks. The property owners indicated that the previous tenants had broken the lease. When vacated by the previous tenant, the property was very messy and required a lot of cleaning of the yard, kitchen, walls, doors, and windows, as well as repair and painting of the walls by the owner. The property had carpet cleaning undertaken, however stains remained on the carpet. The family rented the property, and not long after living in the home they noted an odour and were concerned that the odour may affect their health. This was initially assumed to be mould or damp from recent storms. An inspection was undertaken and there was no evidence of mould or damp indoors, however the inspection noted that the odour increased when windows and doors were shut and was “overwhelming” in rooms with carpet. The source of the odour was unknown and a mould treatment (fogging) was undertaken on the property. The odour remained and the tenants requested further testing, in particular for evidence of illicit drugs. A presumptive test was positive for methamphetamine and they moved out without their possessions prior to further testing being undertaken due to health issues, particularly for the son. Detailed testing was undertaken and the property was found to be contaminated with methamphetamine, assumed to be resulting from use. The tenants went to a tenancy tribunal to attempt to get compensation, however the state in which they reside only requires “clean” premises to be provided to tenants, and the tribunal interpretation of “clean” does not include contamination that is not visible, such as methamphetamine.

CS22: This case study involved the purchase of a suburban home approximately 11 years ago by 2 adults, who then rented the property out for 1 year prior to moving in, after which they discovered the home had been used to grow marijuana. The cleaning of the property for damage caused by hydroponics was covered by the landlord’s insurance. The family moved in and lived at the property for 10 years, with both of their children being born while living at the property. They conducted a number of significant renovations on the property. The children had never been without health problems while living at the property and the owners became suspicious that there might be other drug contamination present. They also discovered that police were aware that the tenant was a drug offender (specifics unknown). The owners had their property tested, which showed that the property was contaminated, so they moved out without their possessions. They regularly visit the property to let the kids play outside and sometimes enter the property. They undertook further testing of the property and possessions, all of which were found to be contaminated. The highest level of contamination was on the air conditioning unit. It is not known whether the property had been used for manufacture at the time when the hydroponics was grown, however the contamination levels that remain in the home are considered too high to be resulting from former use alone. The owners tried to get their insurance company to cover the costs of the methamphetamine contamination from the earlier tenant, however this has been unsuccessful. Since being out of the property the children’s health and behaviour have significantly improved.

CS23: This case study involved a family of 2 adults, 2 children, and their fulltime nanny, who lived in a family-owned suburban home. The father was found to be using methamphetamine over a number of years. The parents were in the process of separation at the time of this study, with the father having moved out of the home. The mother was concerned about the health of the children living in the property, with a number of behavioural issues observed in the children whenever living at the property. The property was tested and was found to be contaminated. Contamination was also found in the boot of the car, suggesting methamphetamine was used in the family car. The levels of contamination reported in the initial testing were found to be higher than would be expected for use alone, however there is no evidence or indication that manufacture ever occurred. The family moved out of the home, leaving possessions behind.

CS24: This case study involved the rental of a suburban home by a family of 2 adults and 3 children for approximately 6 weeks. When they moved into the property, they noticed that the property had been repainted (poorly) and there was a lot of rubbish and building rubble in the garden areas. One of their children, who had pre-existing asthma, was noticeably more unwell when in the home. Discussions with neighbours indicated that the previous tenants may have been using, dealing, or manufacturing drugs. Police were never involved with the property. Based on the suspicious of drug behaviour, they purchased a presumptive test, which returned a positive result. They then organised more detailed testing of the property, which found that the property was contaminated. The testing targeted locations in the home that were not recently painted. The family moved out once contamination was confirmed.

CS25: This case study involved the rental of a house through the public housing authority in a large township by a single older male for approximately 13 months. The first public housing home he rented was found to be located next to a methamphetamine laboratory, which was subsequently seized by police. While living at that property he had a number of issues with vapours and gases from the neighbouring property. After the police seized the laboratory, he was moved to a second rental property in the same town. He noticed an odour in the property and was unwell within days of moving in. Given his experience at the previous property he requested that the property be tested for methamphetamine contamination. Testing was undertaken in the home and it was found to be contaminated in a number of areas, including the bedroom and kitchen. He spent most of the day in the home and reported a number of health issues (including headaches that have required visits to the hospital). He noticed that the health issues resolved whenever he was away from the home for any period of time. He continues to reside in the property at the time of this study, as alternate accommodation is difficult to find.

The 25 case studies included in this study cover a broad range of properties and situations that resulted in exposure to methamphetamine contamination in the property. The duration of exposure in the properties varies significantly. Most had the opportunity to move out as soon as possible after discovering the presence of methamphetamine contamination, however there are some where it was not possible to move out of the home immediately. The broad range of properties outlined above is a snapshot of a complex issue that is often ignored or dismissed, as it has the potential to result in financial, emotional, and physical stress.

### 3.2. Property Details

Table 1 and Table 2 present a summary of the key characteristics of the properties and the contamination status of these properties from each of the case studies. The case studies included in this study are predominantly in urban or suburban areas (22/25 = 88%), most commonly detached houses rather than units (20/25 = 80%), and the majority were rented (18/25 = 72%) rather than owned (7/25 = 28%). Of the properties rented, 33% (6/18) were public or community housing. A wide range of exposure durations occurred, ranging from intermittent visits while moving in to 10 years living in the home. In total, 65 people (37 adults and 28 children) were included in the case studies.

All but one of the case studies relate to properties that were not seized by police as places of manufacture of methamphetamine. Of the properties, 8 (32%) were known or highly suspected to have been used for manufacture only, while 10 (40%) were known to have only been used for the smoking of methamphetamine. In 7 properties (28%), the source of contamination is not clear from the available information and may be from the manufacture or smoking of methamphetamine.

The level of methamphetamine contamination present in the home was characterised on the basis of surface wipe data (presented in Table 2). This information was collected on a property-by-property basis for the purpose of determining the presence of contamination, and in some cases informed remediation actions. As a result, the locations and types of surfaces sampled differed between homes. In addition, the data provided does not always represent surfaces that residents would only come into regular contact with. In some cases, the sampling of surfaces targeted areas where higher levels of contamination may be present, which often include surfaces that are not regularly touched. Such sampling protocols are likely to have biased the results (estimates might be higher than what would be found in more random sampling). Some case studies included a mix of surfaces, while others focused on the surfaces where higher levels were expected.

Exposure in a property may occur via direct contact, resulting in incidental ingestion of residues from hands and items, as well as dermal absorption [24,25,26]. The inhalation of methamphetamine present in dust and in the vapour phase is also expected to occur [1,27]. Hence, all data collected from all surfaces may contribute to the level of exposure. However, as the number of samples and surface types varied between each case study, the average residue level reported in Table 2 should only be considered as indicative.

### 3.3. Data from Individual Participants

From the 25 case studies included in this study, informed consent was obtained from all participants (63 in total), comprising 30 males and 33 females. Of these participants, 34 were adults aged 21 years and older, while 29 were children or adolescents, 22 of whom are aged under 12 years of age. A health survey was completed for all 63 participants, with hair samples collected and analysed from 36 participants. Behavioural assessment forms were completed for 18 of the 29 children and adolescents, all of which returned valid data for evaluation.

In relation to health effects, 6 participants did not report any health effects that specifically related to the time spent in the property. All other participants reported some health effects that were either unique to the time spent living in the property or were exacerbated by living in the property, regardless of the duration of exposure in the property. Of these participants, 67% provided doctors or school reports supporting the health effects or changes in health effects while residing in the property. Where health effects were identified for most participants, these resolved within weeks to months of moving out of the property. One participant, an adolescent, developed a more chronic liver issue that was unchanged after moving out.

The adverse health effects identified have been grouped based on common effects reported: skin irritation or rashes; eye irritation (sore or watering eyes); respiratory effects (persistent cough, asthma or asthma-like symptoms); immune effects (persistent and recurring respiratory infections); sleep issues (difficulty sleeping and unusual dreams); headaches; and behavioural effects (fatigue or tiredness; increased aggression or irritability; depression, anxiety or moodiness; vagueness or not thinking clearly; memory issues). The prevalence of these health effects for the participants included in this study is summarised in Table 3.

Less commonly reported health effects included dental issues, particularly the delayed development of teeth in young children; speech delay in children; weight loss; appetite loss; loss of hair; having extra or excess energy; visual changes; dizziness and nausea; and increased blood pressure and tachycardia.

It is noted that two of the participants involved in this study reported health effects that were so severe that an ambulance was called or hospitalisation was required. This related to headaches in an adult and asthma in a child. In addition, one adult with significant cognitive effects that only occurred while living in the contaminated property had attempted suicide.

Where behavioural assessment forms (BASC-2 or BASC-3) were completed by parents for their children—4 of these reported behaviours that were within the normal range for children of their age, with no indication of any potential clinically significant finding. Most (78%) identified some behavioural indicators that were categorised as “at-risk” or “clinically significant”. One of the children for whom a behavioural assessment form was completed had pre-existing ADHD. The results for this child were excluded from further analyses. For the remaining assessments, behavioural indicators suggested a number of potential clinical issues. These include issues where the behaviours are significantly different to the normal or control population. The results identified mood disorders, principally depressive and anxiety related disorders, as being most common (47%), followed by ADHD (41%) and somatisation and anxiety disorders (23%). This can be compared with the prevalence of anxiety disorders (9.6% for ages 4 to 11 and 7% for ages 12 to 17) and major depressive disorders (1.1% for ages 4–11 years and 5% for ages 12–17 years) in Australian children [28], and the rate of ADHD rates in school-aged children worldwide of around 5% [29], with data from Australia indicating 8.2% for ages 4 to 11 and 6.3% for ages 12 to 17 [28]. Of note is that two of the children, both males who were born and raised in different contaminated properties, have behavioural indicators suggestive of autism spectrum disorder or have been clinically diagnosed with autism spectrum disorder. Based on data from Australia for 2005, autism spectrum disorder affected 0.6% of children aged 6 to 12 years [30].

The analysis of hair samples collected from participants recently exposed in contaminated properties found methamphetamine above the quantitation limit (5 pg/mg) in 20 of the 36 participants sampled. Table 4 presents a summary of the results for each participant for methamphetamine and amphetamine, alongside the average level of methamphetamine contamination identified in the property in which they were living.

The highest levels of methamphetamine and amphetamine in the hair samples were from the single female occupant in case study 16 (CS16F47). This situation involved exposure in a residential unit suspected to have been formerly used for manufacture, but it is also suspected that methamphetamine was continuing to be manufactured in adjacent units, meaning exposure was likely to be from both residual contamination and from active manufacture in an adjacent unit.

The hair analysis data showed higher levels of exposure occurring in younger children, which is consistent with the expectation that younger children will have higher levels of exposures due to more common floor play, poorer washing of hands, and mouthing of hands and objects [8,31]. In addition, young children have more porous hair than adults, which would make the hair more susceptible to environmental contamination [32,33].

## 4. Discussion

### 4.1. Disclosure

In many of the case studies, issues relating to the lack of disclosure in relation to the presence or potential presence of methamphetamine contamination in a property as a result of known or suspected drug activity were raised as significant issue and stressors. A number of individuals renting properties identified that they felt deceived by the actions of owners or real estate agents, and that this deception resulted in their family being placed in a potentially harmful situation. This was particularly evident where health effects were reported in individuals and family members who moved into these properties, where the deception was considered the cause of the health problems occurring in the property. Once the contamination was discovered, many of these individuals reported increased levels of stress and anxiety.

For one property, CS01, where police discovered it was a former methamphetamine laboratory and the local council had issued a clean-up notice, the property was able to be sold without clean-up due to lack of disclosure of the council notice during the property transaction checks. In addition, the local council continued misleading the new owners about the issue, resulting in the family residing at the property for two years. This affected the health of the family and created significant levels of stress and anxiety, as well as financial loss.

Where contaminated properties have not been identified as former drug laboratories by police but there is knowledge of methamphetamine contamination in a property, there are currently no mechanisms available in Australia to ensure that drug contamination is disclosed to current or future tenants or purchasers. In addition, when property purchasers try to obtain information on potential drug history at the property with police, this information is not disclosed due to privacy laws. These barriers to disclosure have the potential to result in public health risks.

### 4.2. Health Effects

This study has identified a range of health effects that are associated with living in methamphetamine-contaminated properties. These are properties that have been contaminated as a result of former manufacture or use. Exposures responsible for these health effects are environmental or third-hand exposures, not use or second-hand exposures during use or manufacture. Further, these exposures are unwitting and range from short-term to chronic.

The identification of health effects relied on self-reported health effects. The focus of the reported health effects related to those that occurred while living in a contaminated home that were exacerbated or different from the participant’s health status before moving into the property. Health effects were reported in 67% of participants. The identified change in health was verified on the basis of medical reports and school reports. The school reports were of particular assistance, as these logged changes in visits to the school nurse, as well as behavioural and academic changes reported by teachers. Many of the identified health effects resolved after moving out of the property, further supporting the idea that the source of the reported health effects related to residing in the contaminated property.

There is no one health effect that all study participants consistently reported. The most commonly reported health effects were behavioural effects or issues (79% of children and 65% of adults); sleep issues, such as difficulty sleeping and unusual dreams (72% of children and 68% of adults); and respiratory effects, such as a persistent cough or asthma-like symptoms (62% of children and 53% of adults).

Skin effects, such as rashes or irritation; and eye effects, such as sore or watering eyes, were also commonly reported in both children (55% for skin and 55% for eye effects) and adults (56% for skin and 59% for eye effects).

Headaches were more commonly reported in adults (47%) than children (7%).

The effects were reported in individuals exposed within the properties over a range of durations, varying between infrequent visits to 10 years. The average duration of exposure for all case studies was 2 years and 2 months, with 47% of individuals exposed for less than 1 year. There were no differences in the frequency or type of health effects reported based solely on the duration of exposure.

Once out of the property, these health effects resolved, as noted in Table 3. Effects on eyes resolving within hours to days, while effects on skin, respiratory system, headaches, and sleep resolving within days to weeks. In some case studies, the resolution of health effects when out of the property while on holidays was one of the first indicators that the home was the cause of their own or family’s health problems, triggering investigation into what was in the home. Behavioural changes also appear to resolve; however, these issues take longer (months to a year). For three participants, depression continued for longer, likely due to prolonged issues in dealing with the contaminated property. In addition, some parents reported ongoing anxiety about their children’s health in the long term.

A common issue relating to the health effects reported for third-hand exposure from methamphetamine-contaminated properties relates to a perception that the health effects commonly reported by individuals are not consistent with those reported from situations with higher levels of exposure, such as during use or exposures by first responders to clandestine drug laboratories. For the key health effects reported in this study, the following provides further supporting evidence of these effects in higher exposure situations.

**Skin problems**: A number of participants involved in this study identified skin rashes, dry skin patches, and irritation or redness as a health issue that related to being exposed in the contaminated property. The prevalence of these effects was similar for both adults (56%) and children (55%). Within the literature, there are numerous reports of skin issues, including burns, associated with manufacture for first responders [34,35,36,37] and children exposed during manufacture [12,38]. The use of methamphetamine results in the picking of skin, and in some cases infections [39]. However, the skin issues reported here are not related to known chemical contact, as would be the case with manufacture, or to skin picking behaviour related to drug-induced psychosis. The skin effects reported in this study related to rashes and hives, and potentially related to contact irritation from residues.

**Eyes**: Sore and watering eyes were commonly reported in this study in both adults (59%) and children (55%). Eye irritation is also commonly reported by first responders to methamphetamine drug laboratories in the US [34,35,36,40]. For methamphetamine drug users, vision loss has been reported, likely due to ischaemic optic neuropathy secondary to methamphetamine-induced vasospasm and methamphetamine-associated vasculitis (or inflammation) [39].

**Respiratory effects**: A number of participants reported respiratory effects, including a persistent cough, asthma, or asthma-like symptoms (which include shortness of breath and wheezing). In this study, these effects were more commonly reported in children (62%) than adults (53%). Respiratory effects are known to be of key concern for individuals exposed during the manufacture and for first responders, including breathlessness, coughing, sore throat and nose, wheezing, and lung damage [34,35,36,37,41,42]. Exposures by first responders have resulted in chronic respiratory effects, including asthma and significantly decreased lung function [41,42,43,44]. Short-term methamphetamine abuse is associated with an increased rate of breathing and constriction in blood vessels, while long-term abuse is associated with decreased lung function and pulmonary hypertension [45].

In addition, the effects of accidental ingestion of methamphetamine by children include acute respiratory problems [46].

In relation to environmental exposures, there are some other reports of respiratory effects. Exposures in former methamphetamine drug laboratories include breathing difficulties reported in a 1 year old child [12] and asthma reported in another child [46] in the US. Respiratory effects (sinus problems in all members of the family and breathing difficulties in a newborn baby) were reported in a family who lived for 5 months in a former methamphetamine drug laboratory in Utah [47].

**Immune issues**: Immune issues, particularly chronic or recurring respiratory and sinus infections, were reported by 32% of adults and 24% of children. It is noted that for some case studies where occupation in the home was for a short period of time, it was more difficult to determine whether the recurring infections were related to exposure in the contaminated property or caused by being in the cold and flu season. However, it was clear from the interviews undertaken that in some case studies participants were more significantly affected by respiratory or sinus infections while living in the contaminated property. Exposures by first responders to methamphetamine laboratories have resulting in effects on the immune system [41,42,43,44].

**Headache**: A number of participants, particularly adults (47%), reported an increase in the number or severity of headaches while living in the contaminated property. For two of the participants from two different case studies, the severity of headaches was so severe that it required hospitalisation and investigation. Other than the changed exposure environment, no other cause of the headaches was identified in either case. On moving out of the property these headaches improved significantly. Headache has been reported as an issue for first responders to methamphetamine laboratories [12,34,35,36,42], as well as properties suspected as former clandestine laboratories [12]. Headaches are also reported in methamphetamine users [48,49].

**Sleep issues**: Difficulty sleeping, including regularly waking up, and unusual dreams were commonly reported in both adults (68%) and children (72%). In a number of case studies, ongoing sleep issues resulted in participants reporting that they were constantly fatigued or tired. When staying out of the contaminated home, most participants reported that their sleep improved within days. This was also reported by parents for children, with sleep improving when out of the home, even where the new location was unfamiliar. Methamphetamine as a single acute dose or repeated administration is known to disrupt sleep in users, even when administered 12 h of more prior to a sleep assessment [50], with low doses used in clinical settings resulting in arousal [49].

**Behavioural and cognitive effects**: A number of participants reported behavioural changes or cognitive effects when living in or visiting the contaminated properties. Overall, this included 65% of adults and 79% of children. A range of different types of behavioural and cognitive effects were reported.

In adults, the most common effects reported related to tiredness and fatigue, which likely resulted from sleep issues, as well as increased moodiness, depression, and anxiety. In some participants with pre-existing depression issues, exposure in the contaminated property significantly exacerbated their symptoms. In relation to the cognitive effects, some participants reported feeling vague and not thinking clearly, as well as memory issues. Effects on memory [42] and mood swings have been reported by first responders to methamphetamine laboratories in the US [34,35]

In children, the most significant effect reported related to increased levels of aggression or irritability, along with increased levels of tiredness and fatigue. These may also be related the sleep issues reported in children living in these properties. For a number of children exposed in these properties, a behavioural assessment was undertaken using a standardised test (BASC-2 or BASC-3). The behavioural issues reported in these case studies are consistent with many of those reported in children removed from methamphetamine drug laboratories [51,52,53,54,55], where there is the assumption that the level of exposure is higher. More specifically, these common behavioural issues include internalising (depression, anxiety, and somatisation) problems, externalising (acting out and hyperactivity) problems, attention problems, and aggressive behaviour [51,52,53,54,55]. However, unlike the behavioural issues reported in children removed from methamphetamine drug laboratories, the effects reported in these case studies are not confounded by other risk factors associated with drug use, criminal behaviour, abuse, and neglect. Another common behavioural issue reported by case study participants was the change in sleep patterns, in particular trouble sleeping. A lack of sleep or significant changes in sleep patterns can also result in changes in behaviour (in particular depression, anxiety, and mood disorders) and a lack of concentration [56].

In general, exposures to amphetamines have been associated with neurochemical changes in areas of the brain that are associated with learning, potentially affecting cognitive function, behaviour, motor activity, and changes in avoidance responses [45]; and physiological, behavioural, and developmental effects, including psychosis, violent behaviour, depression, irritability, hallucinations, mood swings, paranoia, and sleep disorders [45,57,58,59,60,61], with the available data also suggesting cognitive decline or deficits [62,63]. Limited studies on the effects of methamphetamine in adolescents [64] indicate increased levels of depression, anxiety, and risky sexual behaviours, with animal studies indicating impaired cognitive function [65].

**Other effects**: While cardiac effects are considered to be one of the major health effects associated with methamphetamine use [61], effects associated with significant cardiac changes were only reported by one adult participant in this study. One other participant also provided evidence of increased blood pressure and the requirement to double the dose of medication controlling blood pressure while living in the contaminated property. Another commonly reported adverse effect in methamphetamine users relates to dental issues [39]. A child from one of the case studies reported problems with the development of all four molars, which was unusual and could not be explained by their dentist.

An observation provided by one adult participant who was a former drug user is that the effects experienced while living in a contaminated property were consistent with the adverse effects of methamphetamine drug withdrawal. Methamphetamine withdrawal syndrome is characterised by disturbed sleep, including insomnia; depressed mood and anxiety; craving and cognitive impairment; along with agitation, vivid, or unpleasant dreams and reduced energy [49]. A number of these health effects are consistent with those reported in the presented case studies.

### 4.3. Hair Analysis

Not all study participants provided a hair sample for analysis. In a number of cases, this was due to the time period during which they were involved in the study, i.e., they were involved too long after exposure had ceased for any exposure to be able to be detected in the hair (particularly for males with short hair), or the period of exposure in the contaminated property was too short to be able to reliably detect exposure. In some circumstances, hair samples were collected from participants where short-term exposures occurred.

The detection of methamphetamine, and in some cases amphetamine, in the analysis of hair samples from exposed participants, as presented in Table 2, indicates that for some situations environmental exposure was sufficient to be able to be detected in the hair. This is not the case for all situations, as there were a number of participants for whom analysis of a hair sample could not detect methamphetamine.

Amphetamine is the major metabolite of methamphetamine, and the detection of both methamphetamine and amphetamine is generally considered to be indicative of systemic absorption of methamphetamine [66,67], however amphetamine is noted to also be present from the manufacture of methamphetamine in clandestine drug laboratories, which can complicate interpretation of the data.

Amphetamine was detected in the hair analysis of five participants. Where detected, the methamphetamine-to-amphetamine (MA/AMP) ratio was calculated to range between 11 and 23. For CS16F47, methamphetamine was not fully quantified due to the high level detected (>2500 pg/mg), meaning a MA/AMP ratio was not calculated. The range of MA/AMP ratios reported in this study is consistent with the range of 5.7 to 69 and the mean value of 21 reported for hair samples collected from children removed from clandestine drug laboratories [66]. This range was also consistent with the range of 8.6 to 59 and the mean value of 26 recorded for drug-exposed children from the manufacture and use of methamphetamine [67]. For drug users, the MA/AMP ratio is typically around 10 [68], with a reported range of 3 to 50 [69]. This ratio has been found to increase with the duration of drug abuse [70], and presumably environmental exposures.

Based on the MA/AMP ratio from this case study, an estimate of the level of amphetamine that may have been present in the hair of other participants (where methamphetamine was detected) found that the level of AMP in hair for these samples would be below the detection limit of the analysis method.

In relation to the hair washing results, for six participants methamphetamine was detected. The ratio of methamphetamine in the washed hair was in the range of 0.2 to 0.47. Ratios greater than 0.1 have been suggested [71] as indicative of some external contamination. The ratios reported in this study range up to 0.5, a level above which it is suggested that external contamination is significant [71]. This suggests that external contamination of the hair occurred when living in the home, potentially via deposition from the air and direct contact of the hair with hands and surfaces, such as couches and bedding. Where personal possessions were analysed in the case studies included in this study, this data indicated the presence of methamphetamine contamination of possessions brought into the property, supporting the potential for contaminant mobility and external contamination to occur, supporting our previous findings [6].

For adults, methamphetamine was detected in hair in case studies where the mean environmental methamphetamine concentration was in the range 7.8 to 49 µg/100 cm^2^, which predominantly involved properties where clandestine drug manufacture was known or suspected to have occurred. The concentrations of methamphetamine detected in adult hair ranged from 5 pg/mg to 80 pg/mg, with one individual result reported as >2500 pg/mg. The property where the average contamination level was 7.8 µg/100 cm^2^ may have been related to manufacture or use. There were some properties with contamination levels in this range where no detectable levels of methamphetamine were reported in hair. This variability is expected to reflect individual exposure patterns. Higher levels of methamphetamine were reported in hair where the participant was the primary cleaner for the property, spent more time at home, or had a habit of chewing fingernails. The highest level of methamphetamine reported in hair (>2500 pg/mg) related to a situation where there were co-exposures with a neighbouring active manufacturing operation, which also resulted in the highest average level of methamphetamine contamination in the property (49 µg/100 cm^2^).

In relation to children, methamphetamine was detected in hair for case studies where the mean environmental methamphetamine concentration was in the range of 2.8 to 30.7 µg/100 cm^2^. The concentration of methamphetamine detected in the hair of children ranged from 6 to 980 pg/mg. This included properties contaminated as a result of both use and manufacture.

**Figure 1:**
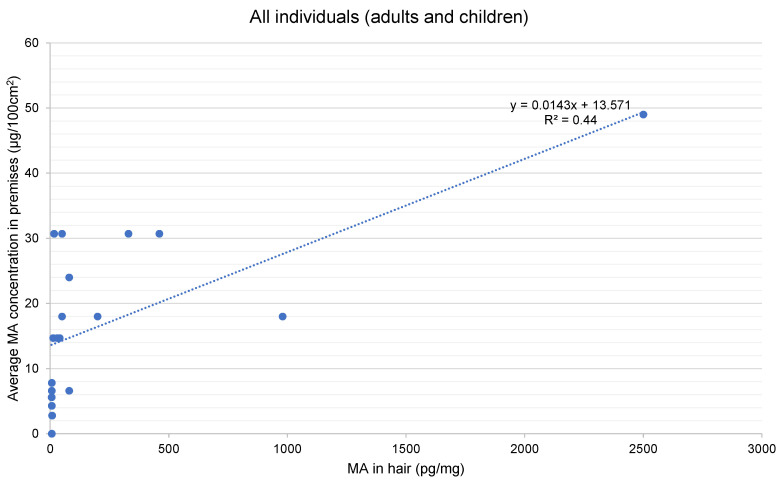

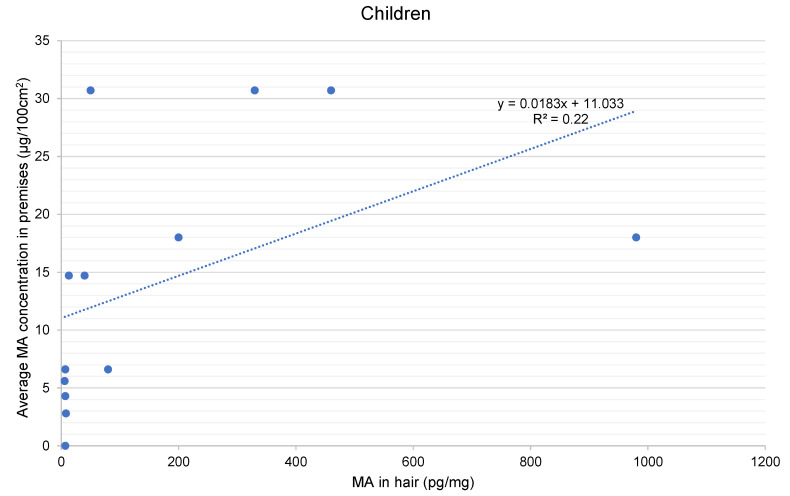
Relationship between environmental methamphetamine (MA) in individual premises and in hair samples collected from individuals exposed in these properties.

Concentrations of methamphetamine detected in hair above the quantitation limit (5 pg/mg) were plotted against the average concentration of methamphetamine reported in the premises these individuals resided in, which are presented in Figure 1 for all individuals (adults and children) and separately for children. While it is noted that there are limitations in the data relating to the characterisation of methamphetamine residues in each property, the figures show a linear relationship between the environmental exposure levels in the premises and the hair concentrations, with the relationship being stronger for all individuals where hair samples were collected (r^2^ of 0.44) than for the samples collected from only the children (r^2^ of 0.22).

**Figure 2:**
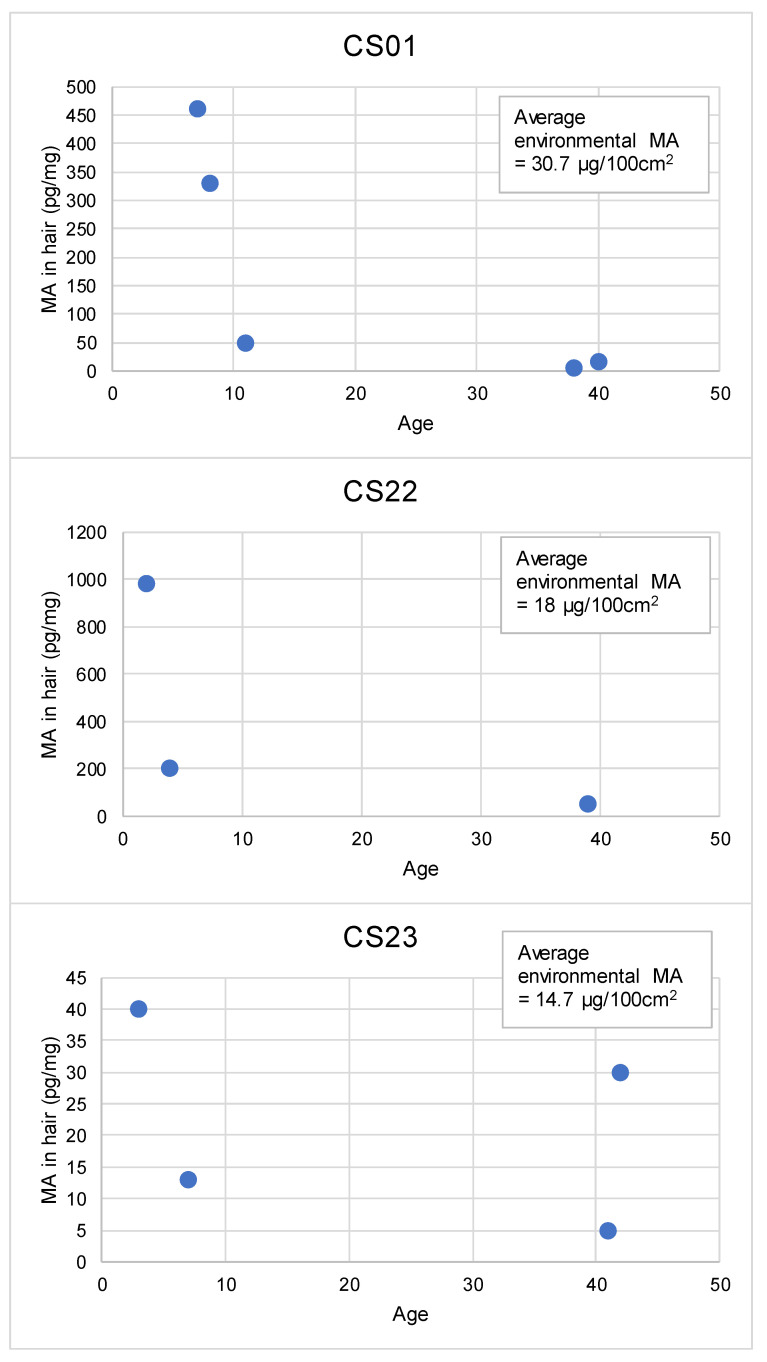
Methamphetamine levels (pg/mg) in hair for families from three case studies (note different scale on the y axes).

**Figure 3:**
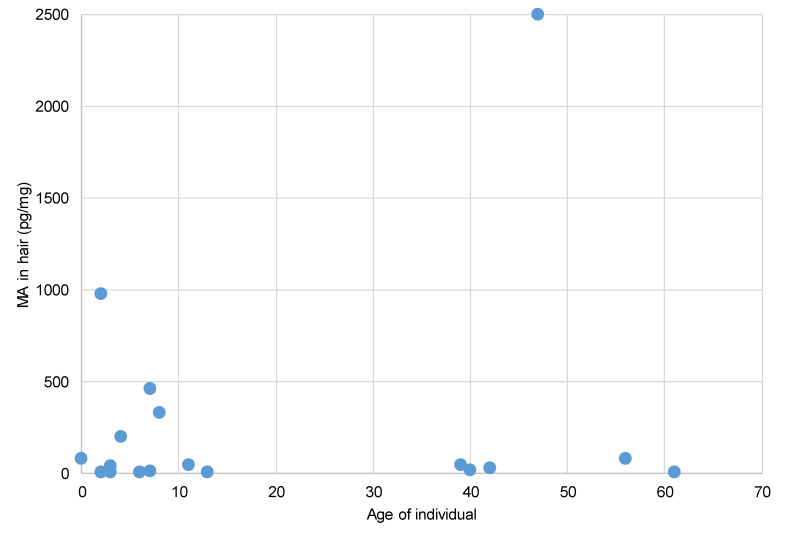
Methamphetamine levels (pg/mg) detected in hair by age for all participants exposed to environmental methamphetamine residues in case studies.

Three of the case studies (CS01, CS22, and CS23) included families exposed in contaminated properties where methamphetamine was detected in hair in both adults and young children. The concentrations of methamphetamine reported in these case studies are shown in Figure 2. For these case studies, the level of methamphetamine reported in hair follows a pattern consistent with what is expected for environmental exposures, with higher levels reported in younger children who have higher levels of exposure to surfaces, objects, and materials in the home. The exception is CS23, where one of the adults, the children’s nanny, had higher levels of methamphetamine than the mother. This may be due to exposures that occur during close play with young children. The highest levels were reported in the youngest children. Figure 3 shows the concentrations of methamphetamine by age in all participants from exposure to methamphetamine residues in all properties, which shows the decreasing levels of methamphetamine detected in hair with increasing age of the participants exposed.

The levels of methamphetamine found in hair in this study, particularly in the children’s hair from CS01 and CS22, were at the lower end of the range reported in adults and children from properties where methamphetamine was actively used or manufactured [66,67] and the range reported for adult drug users [72,73]. The ranges of methamphetamine reported in hair in these studies were 100–131,000 pg/mg for children and 100–128,100 pg/mg for adults, noting that a reporting cut-off of 100 pg/mg was adopted in these studies. A study of passive exposures by children in families where one or both parents used drugs reported detectable levels of levels of amphetamines (MA + AMP) in 80% of the situations where these drugs were used by the parents, with concentrations of 140 to 890 pg/mg reported in the hair of children aged 7 to 10 years [33]. These studies also showed that the higher levels of methamphetamine in hair were reported for children aged 5 years and younger, with a decreasing trend with increasing age. This is consistent with the behaviours of young children, including increased floor play, a higher potential for touching walls and other surfaces in the home, a higher potential for placing hands and objects in the mouth, less frequent washing of hands prior to eating, and more time spent indoors, all of which may result in higher levels of exposure. It is also suggested that the finer and more porous nature of the hair of children aged up to around 3 years may facilitate incorporation from external contamination [33]. Where children are exposed to residues in contaminated properties, as is the case for this study, incorporation from external contamination is expected to be a contributing factor in the levels reported in hair.

### 4.4. Use vs. Manufacture

While some data are available relating to health effects and exposures that occur during and post manufacture of methamphetamine, data are lacking in relation to health effects and exposures that occur as a result of residues derived from the smoking of methamphetamine.

This study has included data collected from properties and participants exposed to methamphetamine residues as a result of either known or suspected manufacture or use alone. For some case studies, the source of contamination was not clear, and hence manufacture and use were both been considered. The information provided on the likely source came from police reports, media reports of police activity, observations of property owners in relation to evidence of manufacture or use, and information from neighbours on the behaviour of previous occupants and presence of unusual odours. As a result, while this evidence has been used to categorise the contamination as being derived from manufacture or use, it cannot be confirmed. Both manufacture and use by previous occupants are illegal activities, and hence it is not possible to be more accurate in determining the most likely source of contamination.

**Figure 4:**
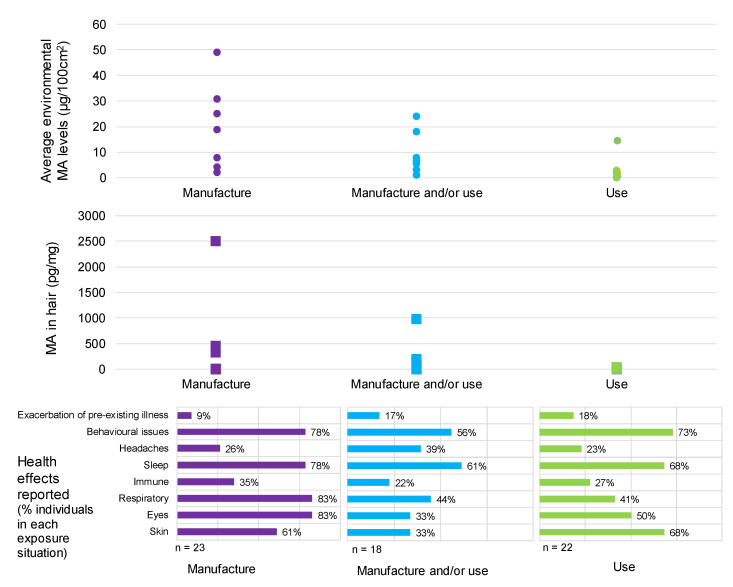
Summary of exposure and health data by exposure situation for manufacture and use.

Figure 4 presents a summary of the data collected from contaminated premises and participants, separated into the three different exposure situations: manufacture; manufacture and/or use; and use. This figure shows the range of average methamphetamine environmental levels reported in properties—the results of hair samples collected encompass higher levels for manufacture compared with use. Despite the reduced range of methamphetamine environmental levels reported in properties contaminated from use, it is not possible to preclude contamination from manufacture simply on the basis of the lower levels of environmental contamination reported, as the lower levels of environmental contamination were also reported in properties where manufacture was known, suspected, or cannot be precluded. The presence of higher average methamphetamine environmental levels could be used to identify properties associated with former manufacture.

Similarly, the range of methamphetamine concentrations reported in hair was lower for participants exposed in properties contaminated from use. However, lower levels, including levels below the detection limit, were also reported in properties where contamination from manufacture was known, suspected, or could not be precluded.

In terms of health effects reported by participants exposed in contaminated properties, these were consistently reported regardless of the source of the methamphetamine contamination. The only key difference in the health effects reported was a higher prevalence of eye and respiratory issues in properties known or suspected to have been contaminated from manufacture. The presence of contamination and public health risks from use are generally poorly addressed in guidelines, which predominantly focus on contamination from manufacture. The data presented in this study indicate that contamination from exposure alone is a potential significant public health issue.

### 4.5. Guidelines

Guidelines for the assessment of methamphetamine-contaminated properties are currently available in Australia and internationally. In Australia, guidelines are available for methamphetamine in residential homes, as well as commercial or industrial premises [74]. These are risk-based guidelines that adopt a range of assumptions about the way in which people are exposed to methamphetamine in a property via dermal absorption and ingestion only, which involve published quantitative toxicity reference values [75]. As a result, the guidelines are intended to be protective of the health of individuals exposed in methamphetamine-contaminated properties. Any such guidelines should have a margin of safety such that observable health effects would not be expected with exposure in premises where methamphetamine contamination is at or just above the guideline.

The data presented here cannot be used to identify an average level of environmental methamphetamine exposure below which no health effects are reported. This is principally due to the reporting of health effects associated with exposure in the contaminated properties over a wide range of contamination levels, including case studies where the average methamphetamine level was less than or close to the current Australian residential guideline of 0.5 µg/100 cm^2^. There were three case studies where the average environmental methamphetamine levels were below this guideline.

CS15 was exposed to the lowest average environmental methamphetamine contamination level of 0.063 µg/100 cm^2^. Health effects were reported in this case study for both participants—an adult with a pre-existing illness and a 13-year-old child with pre-existing asthma. The reported health effects included skin and eye irritation, persistent cough, exacerbation of asthma, sleep issues, headaches, and behavioural changes, including increased depression and anxiety in the child and vagueness in the adult. In this case study, medical and school reports were provided showing evidence of the health effects reported during occupancy of the property. The property was remediated by the owner despite the environmental methamphetamine levels being below the Australian guideline for residential properties. Following remediation, the reported health effects resolved as the family continued to live in the property post-remediation.

CS06 was exposed to an average environmental methamphetamine contamination level of 0.31 µg/100 cm^2^. This case study included one adult participant for whom health effects were reported. These health effects included skin and eye irritation, persistent cough, depression, fatigue and vagueness, dizziness, nausea, and weight loss. Medical reports were provided to support the reported health effects. Apart from depression, the health effects resolved on moving out of the property. The persistent depression was likely exacerbated by ongoing issues related to resolving financial losses that occurred when living in and moving out of the contaminated property.

CS21 was exposed to an average environmental methamphetamine contamination level of 0.36 µg/100 cm^2^. This case study included one 16-year-old participant for whom health effects were reported, which included skin rashes, persistent cough, and vagueness. Medical reports were provided to support the reported health effects. These health effects resolved after moving out of the property.

None of the three case studies discussed above included analysis of a hair sample.

For the case studies where hair analysis was undertaken, the lowest average environmental methamphetamine level that resulted in a detection of methamphetamine in hair was CS05. This property had an average environmental methamphetamine level of 2.8 µg/100 cm^2^ and trace levels of methamphetamine were reported in the 13-year-old participant residing in the property. This property is suspected to have been contaminated as a result of use only. Other case studies with lower levels of average environmental methamphetamine that were up to approximately 10 times above the Australian guideline, CS18 and CS12, also reported trace levels of methamphetamine in the hair of children residing in these properties. Methamphetamine was not detected in the hair samples collected from adults in these properties. These data demonstrate that environmental methamphetamine exposure and intake is occurring at properties more commonly considered to have low levels of methamphetamine contamination.

One of the key issues identified with the data reported in this study is the use of environmental methamphetamine data collected by other parties or investigators, which resulted in the sampling of different surfaces and materials in each case study. Hence, there is the potential that higher levels of methamphetamine contamination may be present in the properties in unsampled areas where participants may be regularly exposed. This is, however, a real issue, as decisions in relation to contamination levels and remediation are based on the same types of assessments undertaken by investigators that were used in this study. While it is accepted that some of these assessments will have considered property-specific aspects, the variability in the numbers of samples collected and the types of materials sampled is significant. This complicates the review of the data presented in this study; however, this study has relied on the same data that participants, regulators, and industry would rely on when making decisions about the property. This suggests that more robust training may be required for investigators to minimise the potential to underestimate, or even overestimate, environmental methamphetamine levels in properties [76].

### 4.6. Study Limitations

This study has included data from opportunistic case studies only. The issues addressed relate to contamination from former illegal activities, and hence information is limited or unavailable in relation to the source of contamination (manufacture or use) and locations of the properties where such activities may have occurred.

Data provided in relation to environmental methamphetamine levels in the properties were from investigations undertaken by others for the purpose of characterising contamination. As a result, the data were mixed in terms of those collecting the samples, the number of samples collected, as well as the surfaces that were sampled within each property. While these are the same data that are relied on for making decisions about the contamination status of a property, the variability in the sampling makes it more difficult to compare results between properties.

This study relied on self-reported health effects for participants. This information specifically focused on health issues that were different while residing in the contaminated property, rather than just the participants’ current health status. Participants were asked to provide health information in relation to exposures prior to their knowledge or confirmation that the property was contaminated. In addition, this study obtained or cited medical and school reports supporting these observations for 67% of the participants. While all attempts were made to limit reporting bias from participants, it is expected that this will be present. It is also not possible to preclude other issues inside each property that may have contributed to or affected participant health.

## 5. Conclusions

This study has identified a range of health effects that occur while residing in contaminated properties, which include behavioural effects or issues, sleep issues, respiratory effects, skin and eye effects, and headaches. In addition, methamphetamine was also detected in the analysis of hair samples collected from a number of individuals, including children exposed at these properties. In general, higher levels of methamphetamine were reported in hair samples from individuals exposed in properties with higher levels of environmental methamphetamine contamination. The exposures and health effects reported occurred following exposure to properties contaminated as a result of known or suspected manufacture or use over a wide range of environmental methamphetamine levels in the property, which included levels close to the current Australian guideline of 0.5 µg/100 cm^2^. There were no discernible differences in exposures or health effects reported in properties contaminated from former manufacture or use.

The characterisation of contamination at these types of properties is complex, as are the situations in which exposure has occurred in each of the case studies evaluated, with the majority of these properties not being known to have had a drug history prior to occupancy. This study has demonstrated that these properties have the potential to be a significant public health risk. It is, therefore, important that these properties are properly identified and cleaned up and that procedures are developed to ensure that clean-up occurs. Additionally, properties known to be contaminated but not cleaned up need to be disclosed to future occupants.

## Figures and Tables

**Table 1:** Summary of key aspects associated with case studies.

Case Study	General Location		Type of Property		Ownership			Occupants		Duration of Time in Property
	R	U	H	A	O	T	PH	Adults	Children	
CS01	y		y		y			2	3	1.5 years
CS02		y	y			y		1	2	2 years
CS03		y	y		y			1		8 months
CS04		y	y			y		2	2	5 weeks
CS05		y	y			y	y	2	1	3 years
CS06		y	y			y	y	1		7 years
CS07		y	y			y		1	1	2 years
CS08		y	y		y			2	3	4 months
CS09		y		y		y		2		5 months
CS10	y			y		y	y	1		3 years
CS11		y	y			y		1		7 months
CS12		y	y			y		2	3	4.5 months
CS13		y	y			y	y	1		Regular short visits
CS14		y		y		y			1	10 days per fortnight over 2 years
CS15		y		y		y	y	1	1	3 years
CS16		y		y		y		1		8 months
CS17		y	y			y		1		5 months
CS18		y	y			y		2	2	8 months
CS19		y	y		y			2	2	8 years
CS20	y		y		y			2		2 years—few days at a time
CS21		y	y			y			1	4 weeks
CS22		y	y		y			2	2	10 years
CS23		y	y		y			2	2	6 years
CS24		y	y			y		1	3	6 weeks
CS25		y	y			y	y	1		13 months
N =	3:22		20:5		7	18	6	34:29		

R = Rural; U = Urban; H = House; A = Apartment or unit; O = Own; T = Tenant or rental; PH = Public housing (or similar).

**Table 2:** Contamination status of properties included in case studies.

Case Study	Activity Likely to Have Resulted in Contamination		Contamination Status of Methamphetamine, Based on Wipe Sample Results (µg/100 cm^2^)		
	Manufacture	Use	Minimum	Maximum	Average *
CS01	y (police seized)		11	107	30.7
CS02	y		<0.02	42	2.2
CS03	y		0.01	>10	NA
CS04	y, suspected		7.3	8.3	7.8
CS05		y	<0.03	20.7	2.8
CS06		y	<0.03	1.32	0.31
CS07		y	0.02	4.4	0.96
CS08		y	0.1	5.7	2.2
CS09	potential	potential	0.12	53	3.1
CS10	y, suspected		0.69	>100	25
CS11	potential	potential	0.19	42.9	7.8
CS12	potential	potential	3.5	7.8	5.6
CS13		y	<0.02	20	2
CS14		y	NA	NA	NA
CS15		y	<0.02	0.13	0.06
CS16	y		4.1	107	49
CS17	potential	y	5.34	45.09	24
CS18	y		0.32	9.9	4.3
CS19	y		<0.02	245	18.8
CS20		y	0.05	31	2.3
CS21		y	<0.02	2	0.34
CS22	potential	potential	0.38	250	18
CS23		y	0.18	125	14.7
CS24	potential	potential	0.03	25	6.6
CS25	potential	potential	<0.02	3.5	1.2
Manufactured—Yes	7				
Manufactured—Suspected	2				
Manufactured—Potential	7				
Manufactured—No	10				
Use—Yes		11			
Use—Potential		6			
Use—No		8			
Manufactured (Potential) + Use	1				
Manufactured (Potential) + Use (Potential)	6				

* Average level of contamination based on an arithmetic average of all samples collected, from all locations sampled in each property. It is noted that the sample locations differed for each property. Where contamination was reported as not detected, the analytical limit of reporting as stated by the laboratory was used. NA = data not available. For CS03, the available wipe sample results were provided as a minimum and maximum, and due to the historical nature of the contamination issues further testing was not possible. For CS14, the contamination status of the property was not available as access to the property was not possible.

**Table 3:** Prevalence of health effects reported by study participants when exposed in contaminated properties.

Health Effect Reported	Number of Participants Reporting Health Effect as Number (% All Participants)		Resolution of Health Effects Once Out of the Property
	Children and Adolescents [*n* = 29]	Adults, 21 Years and Older [*n* = 34]	
Skin (rashes, irritation)	16 (55%)	19 (56%)	Yes—within days to weeks
Eyes (sore, watering)	16 (55%)	20 (59%)	Yes—within hours to days
**Respiratory—total**	**18 (62%)**	**18 (53%)**	
- Persistent cough	16 (55%)	16 (47%)	Yes—within days to weeks
- Asthma or asthma-like symptoms	10 (34%)	10 (29%)	Yes—within days to weeks
Immune (chronic or constant infections—respiratory or sinus)	7 (24%)	11 (32%)	Yes—within weeks
Headache	2 (7%)	16 (47%)	Yes—within days to weeks
**Sleep—total**	**21 (72%)**	**23 (68%)**	
- Difficulty sleeping	18 (62%)	21 (62%)	Yes—within days to weeks
- Unusual dreams	12 (41%)	10 (29%)	Yes—within days to weeks
**Behavioural and cognitive—total**	**23 (79%)**	**22 (65%)**	Most resolved within a month to a year. For 3 participants, depression continued for a longer time, likely due to prolonged issues in dealing with the contaminated property. In addition, for some parents there is ongoing anxiety about their children’s health in the long term.
- Fatigue or tiredness	8 (28%)	13 (38%)	
- Increased aggression or irritability	16 (55%)	5 (15%)	
- Moodiness, depression, anxiety	5 (17%)	13 (38%)	
- Vagueness or not thinking clearly	5 (17%)	10 (29%)	
- Memory issues	0	6 (18%)	
Exacerbation of pre-existing conditions	2 (7%)	7 (21%)	Yes—within months

**Table 4:** Results of hair analysis for participants.

Sample Code *	Age (Years)	Hair Analysis Results (pg/mg)				Average Level of MA Contamination in Property ** (µg/100 cm^2^)	Likely Source of Contamination
		Hair Matrix		Hair Wash			
		MA	AMP	MA	AMP		
CS01F40	40	17	<5	8	<5	30.7	M
CS01M38	38	5	<5	<5	<5	30.7	M
CS01F11	11	50	<5	<5	<5	30.7	M
CS01M8	8	330	16	<5	<5	30.7	M
CS01M7	7	460	20	<5	<5	30.7	M
CS04F2	2	<5	<5	<5	<5	7.8	M
CS05F13	13	8	<5	<5	<5	2.8	U
CS08M11	11	<5	<5	<5	<5	2.2	U
CS08M8	8	<5	<5	<5	<5	2.2	U
CS08F6	6	<5	<5	<5	<5	2.2	U
CS09F30	30	<5	<5	<5	<5	3.1	M and/or U
CS10F44	44	<5	<5	<5	<5	25	M
CS11F61	61	7	<5	<5	<5	7.8	M and/or U
CS12M10	10	<5	<5	<5	<5	5.6	M and/or U
CS12M6	6	<5	<5	<5	<5	5.6	M and/or U
CS12M3	3	6	<5	<5	<5	5.6	M and/or U
CS14M2	2	7	<5	<5	<5	NA	U
CS16F47	47	>2500	590	710	100	49	M
CS17M56	56	80	7	<5	<5	24	M and/or U
CS18F47	47	<5	<5	<5	<5	4.3	M
CS18M13	13	7	<5	<5	<5	4.3	M
CS19F44	44	<5	<5	<5	<5	18.8	M
CS19M51	51	<5	<5	<5	<5	18.8	M
CS19M7	7	<5	<5	<5	<5	18.8	M
CS19F4	4	<5	<5	<5	<5	18.8	M
CS22F39	39	50	<5	11	<5	18	M and/or U
CS22F4	4	200	15	40	<5	18	M and/or U
CS22M2	2	980	50	280	17	18	M and/or U
CS23F41	41	<5	<5	<5	<5	14.7	U
CS23F42	42	30	<5	<5	<5	14.7	U
CS23F7	7	13	<5	<5	<5	14.7	U
CS23F3	3	40	<5	<5	<5	14.7	U
CS24F8	8	<5	<5	<5	<5	6.6	M and/or U
CS24M6	6	7	<5	<5	<5	6.6	M and/or U
CS24M0	0.66	80	<5	16	<5	6.6	M and/or U
CS25M63	63	<5	<5	<5	<5	1.2	M and/or U

M = environmental contamination as a result of known or suspected manufacture; U = environmental contamination as a result of known or suspected use (smoking). * = Sample code relates to the case study number, gender of participant and age of participant, e.g., CS12M3 is a participant from CS12, is male, and aged 3 at the time of the study. ** = Indicative average level of MA in properties, noting that the data for each property are variable in terms of the number of samples collected and the type of surfaces sampled.
